# Quality of Life and Nutritional Status of the Geriatric Population of the South-Central Part of Nepal

**DOI:** 10.1155/2021/6621278

**Published:** 2021-04-30

**Authors:** Sabita Sharma, Dipendra Kumar Yadav, Isha Karmacharya, Raju Pandey

**Affiliations:** ^1^School of Health and Allied Sciences, Faculty of Health Sciences, Pokhara University, Pokhara, Nepal; ^2^CIST College, Kathmandu, Nepal

## Abstract

**Background:**

The main objective of the study was to assess the nutritional status and quality of life in the geriatric population of Lahan municipality of Siraha district.

**Methods:**

A cross-sectional analytical study was conducted in Lahan municipality of Siraha district from June to December 2017. The Mini-Nutritional Assessment tool was used to investigate the nutritional status, and World Health Organization Quality of Life-OLD questionnaires were used to assess the quality of life among geriatric population.

**Result:**

Out of the total participants, one-third (45.7%) of the participants were at risk of malnutrition and 19.8% were malnourished while 34.5% had normal nutritional status. It was seen that 48.2% of participants had good quality of life whereas 51.8% of them had poor quality of life. There was a significant association between nutritional status and quality of life in the elderly population.

**Conclusion:**

The findings showed the need for active ageing interventions to improve the nutritional status and quality of life of elders at the community settings. Proper attention should be focused on elders' nutrition to reduce the observed prevalence of malnutrition, and focus should be given on the nutrition status that leads to improve the quality of life of elders.

## 1. Introduction

Aging is a complex process with changes in physiological, psychological, and social factors that may impact nutritional status [1]. According to the census report of Nepal, the population of the elderly, defined by the Nepali Senior Citizens Act as persons 60 years of age and above [2], grew from 1.5 million in 2001 to 2.2 million in 2011 [3]. Adequate diet and nutritional status are important health determinants for quality life at this stage [4].

Malnutrition is a frequent syndrome in the elderly and refers to faulty or inadequate nutritional status and undernourishment characterized by insufficient dietary intake, poor appetite, and muscle wasting and weight loss [5]. In Nepal, poverty and inadequate government social security benefits, combined with the lack of knowledge about nutrition, further contribute to the vulnerability of the elderly to malnutrition [3]. The Mini-Nutritional Assessment (MNA) is a validated nutrition screening and assessment tool which was developed using data generated from geriatric patients in the United States and Europe and validated with clinical data of Caucasian populations. This tool is widely used and accepted and specifically developed to evaluate the nutritional status of the elderly who are malnourished or at risk of malnutrition [6].

Quality of life is an individual's perception of their position in life within the context of the culture and value systems during which they live and in reference to their goals, expectations, standards, and concerns. Quality of life is important for elderly people as it decreases with increasing age [7]. The WHO Quality of Life Assessment for Older Adults (WHOQOL-OLD) was originally developed by the WHOQOL group for the investigation of quality of life in older adults. A poor QoL in older persons might reflect health problems relating malnutrition, disability, and dependency. Therefore, this relationship should be investigated, prevented, and susceptible to improvement [8].

One in six persons globally was affected by malnutrition in 2015 [3]. Quality of life is reduced in the elderly at risk of malnutrition [9]. The prevalence of malnutrition in Europe and North America is 1–15% in noninstitutionalized older adults [10]. Till now, the information regarding the nutritional status and quality of life of the elderly in Nepal is very minimal, while some studies conducted showed only the prevalence of malnutrition which is 15.5% to 31% [3]. Thus, this study was designed to assess the quality of life and the nutritional status of the geriatric population in Lahan municipality, as well as to identify the association between nutritional status and quality of life.

## 2. Methods

### 2.1. Study Design and Setting

This study was a community-based cross-sectional analytical study conducted in Lahan municipality of Siraha district of Nepal from June to December 2017. Lahan municipality lies in the Terai of Nepal that is rich in food availability. Few studies have been conducted in the area, particularly focusing only on nutritional status of children and women [11, 12]. Little significance is given on geriatric health, nutrition, and quality of life.

### 2.2. Study Population and Sampling

The geriatric population aged 60 years and above in Lahan municipality was enrolled with total sample size 328 using the probability proportionate size technique, assuming the prevalence of malnutrition among the geriatric population to be 31% [13] with an error of five percent and 95 percent confidence limit. In the first stage of the sampling, about 42% of total 24 wards, i.e., 10 wards, were selected randomly by the lottery method. The center of each ward was identified with the help of Google Maps version 9.73.3. The first household was selected by spinning a pencil in the direction shown by the tip of the pencil. From the selected house, eligibility of participants was assessed, and from each household, only one eligible participant was surveyed. In cases where none of the household members was considered eligible, a participant from the adjacent household with the closest front door was approached. In case of multiple eligible participants in the same household, only one was selected randomly by the lottery method. To be eligible, participants had to be 60 and above and resident of the study area for at least a year.

### 2.3. Study Tools and Data Collection

The Mini-Nutritional Assessment (MNA) [6] tool developed by the Nestle Nutrition Institute was adapted to measure nutritional status, whereas the WHOOLD-QOL Questionnaire [7] was used to identify the quality of life of the study population. A face-to-face interview with the participants was conducted.

Tools were translated in Nepali language and translated back by a translator. Pretesting was conducted among 35 elderly people matching the eligibility criteria in Pokhara Metropolitan. Minor typing mistakes were omitted, and the questionnaire was revised.

### 2.4. Study Variables

#### 2.4.1. Dependent Variables

The nutritional status is categorized between three groups according to the coverage score in the MNA screening tool. Nutritional status assessment was performed using the MNA tool with 18 questions, which consist of anthropometric assessment: height and weight (BMI) or calf circumferences; general assessment: lifestyle, medication, and mobility; dietary assessment: food and fluid intake, autonomy of feeding, and number of meals; and subjective assessment: self-perception about health and nutrition [14].

The nutrition status score was categorized as follows: elderly with scores 0–7 were considered as malnourished; elderly with scores 8–11 were considered as at risk of malnutrition; and elderly with scores 12–14 were considered as having normal nutritional status. The nutritional status was further categorized into binary categories with the code “0” as normal and “1” as at risk/malnourished.

The quality of life is measured using the WHOQOL-OLD questionnaire in which there are 24 items covering six facets: sensory abilities; autonomy; past, present, and future activities; social participation; death and dying; and intimacy. Scores for each domain were calculated by adding the value of each item. The domain scores were transformed to a 0–100 scale. A normality test was performed, and the median score was taken to categorize quality of life. Those who scored below 37 values of quality of life were considered to have poor QOL, while those who scored equal and above 37 values were considered as having good QOL.

#### 2.4.2. Independent Variables

Attributes such as age in years, gender, either male or female, religion, belonging to a Hindu family or others, ethnicity, either disadvantaged non-Dalit or others, educational status, either literate or illiterate, and past occupation, either paid works or nonpaid works, were considered as independent variables.

### 2.5. Statistical Analyses

The data were primarily entered in Epi data version 3.1 and analyzed using SPSS 16.0 version. Data were summarized in terms of frequencies (proportion, percentage, mean, or median). The Kolmogorov–Smirnov test was applied to test normality of the data. Among the variables that were normally distributed, mean (standard deviation) was applied; otherwise, median (interquartile) was used.

Dependent variable was categorial. So, associations were tested using Pearson's chi-square test and logistic regression taking 95% confidence interval.

### 2.6. Ethical Considerations

The Institutional Review Committee of Pokhara University approved this study. The participants were informed about the objectives of the study and that participation was voluntary. Confidentiality was maintained, while written consent was taken from each respondent prior to data collection.

## 3. Results

### 3.1. Sociodemographic Characteristics

Among the 328 participants, 46.0% were between 60 and 67 years of age while others were above 67 years of age, with mean age 68.52 ± 6.99 years. More than half (57.9%) were males. More than ninety percent of the participants were Hindu, and those who were disadvantaged non-Dalit by ethnicity were 61.9%. 35.7% of the participants were illiterate, followed by 23.2% with nonformal education, while only 1.5% had education of the bachelor and above level. About 44% of the participants previously worked in the agriculture sector, followed by 18.3% doing business, 14.9% who had job, and 8.8% respondents engaged in daily-wage work (Table 1).

### 3.2. Nutritional Status of the Participants

Nutritional status assessment was performed by using the MNA scale with seven questions, which included anthropometric assessment and general assessment. Among 328 participants, 113 (34.5%) had normal nutritional status; 150 (45.7%) were at risk of malnutrition; and 65 (19.8%) were in the range of malnourished in MNA scores.

The age group of 68 and above (94.5%) was found to be at risk or malnourished than those of the age 60–67 years (37.0%). Malnutrition was seen slightly higher in females (68.8%) than males (63.2%). In addition, the majority of participants from the disadvantaged non-Dalit caste group were at risk of malnutrition or malnourished (67.5%). Furthermore, illiterates (76.2%) were malnourished in comparison to literate ones (59.2%). Majority of participants from the nonpaid work group were at risk of malnutrition or malnourished (70.5%).

Age (*p* < 0.001), religion (*p* = 0.022), educational status (0.002), and past occupation (*p* = 0.026) were associated with the nutritional status of the study population (Table 2).

In the multivariable binary logistic regression model, age was significantly associated with nutritional status (Table 3). The respondents 67 years old and above were about 28 times more as likely to be at risk of malnutrition or malnourished as the 60–67 years age group (OR: 28.34, 95% CI: 13.35–60.18). However, religion, educational status, and past occupation were not associated to nutritional status after adjusting the covariates of nutritional status in logistic regression (Table 3).

### 3.3. Descriptive Statistics of Transformed Score of QOL

As per the WHOQOL-OLD manual, raw scores for the facets were calculated by adding values of single items and were transformed on the scale ranging from 0 to 100, where 100 was the highest and 0 was the lowest QOL. Mean score of each facet and the total scores were calculated. Among the elders, facetwise mean and standard deviation score for sensory abilities was 49.25 ± 20.28, for autonomy, it was 37.74 ± 20.95, for past, present, and future abilities, it was 32.81 ± 20.95, for social participation, it was 31.40 ± 18.32, for death and dying, it was 34.79 ± 21.68, and for intimacy, it was 31.00 ± 17.70, and the mean score of overall QoL was 36.167 ± 19.667 (Table 4).

### 3.4. Categories of Quality of Life Based on Scores

Elders with the total mean score of 37 and above were classified as having good QOL and less than 37 as having poor QOL. Out of total 328 respondents who were interviewed, 158 (48.2%) had good quality of life whereas 170 (51.8%) had poor quality of life according to the raw scores obtained by adding all the facets scores of WHOQOL-OLD questionnaires (Table 5).

Among different demographic variables, age and educational status of participants were associated with quality of life. Gender, ethnicity, religion, and previous occupation of the respondent had no association with quality of life (Table 6).

### 3.5. Nutritional Status and Quality of Life

According to Pearson's correlation coefficient, the nutritional status was significantly associated with QOL domains with (*p* < 0.001). So, the elderly who had impaired nutritional status, compared to the normal nourished elderly, was significantly differed in the QOL domain. Also, the result mentioned above states that as the nutritional status of elders declines, their quality of life also becomes poorer (Table 7).

## 4. Discussion

The study performed reveals that, among the elder people living in Lahan municipality, only 34.3% had normal nutritional status according to their MNA scores. Initial findings were presented in the fourth national summit of health and population scientists in Nepal [15] and after rigorous discussion that help the finalization of the research findings.

Age is significantly associated with nutritional status, which states that nutritional status decreases with increasing age. Similarly, educational status and past occupation were associated with nutritional status. In case of quality of life of geriatric population, 48.2% had good quality of life while 51.8% had poor quality of life. Factors such as age, ethnicity, religion, educational status, and past occupation showed a positive association with quality of life. Nutritional status was associated with overall QOL.

Nearly half of the elders (45.7%) were found to be at risk of malnutrition among the sampled elderly population in Lahan municipality which was near to the findings among the elderly of Dakshinkali VDC of Nepal [3]. In the study reported by Naidoo among community-dwelling elders in KwaZulu-Natal, South Africa, a similar finding was reported at risk of malnutrition [16]. In the present study, the percentage of malnourished was found to be 19.1 which is near to the findings of the study conducted by different researchers [14].

In addition, a statistically significant association was observed between the age group of people, religion, educational status, and past occupation of the participants, and nutritional status with *p* value <0.001, 0.022, 0.002, and 0.026, respectively, in the present study. Similarly, an association between age groups and educational status was shown in the studies conducted in Nepal [3], India [17, 18], and Tanzania [1]. But, a negative association of sex and nutritional status was observed in the present study which is contrary to the few studies conducted in India and Kuala Lumpur, which shows the positive association of sex and nutritional status. The difference in findings may be due to the difference in status of free-living old-age people of Lahan municipality. The study also presents no association of nutritional status with ethnicity and religion which was shown by Ghimire et al. in the study conducted in Nepal. This may be due to the presence of more number of people from the same caste group and religion.

The study conducted in Pharping, Nepal, Spain [19], and Iran [20] showed that people with poor economic status and educational status were at higher risk of being malnourished. This is the fact which shows that socioeconomic and educational difference affects the nutritional status of individuals. Similarly, past occupations of the elders also affect their nutritional status. Moreover, several factors appear to contribute to the nutritional status of the geriatric population evidenced with aging. Factors such as low food intake, weight loss, psychological stress, and mobility also affect the nutritional status of elders as shown in the study performed by different researchers [9, 21].

Different sociodemographic factors such as age, ethnicity, religion, marital status, educational status, and past occupation are positively associated with an individual's quality of life, which was also shown in various studies conducted in different countries including Nepal. In the present study, it is seen that both genders are equally susceptible to have poor quality of life with increasing age, similar to some studies, which revealed that quality of life decreases with increasing age [22, 23].

In addition, this study showed that people with lower level of education had poorer quality of life than those with higher level of education, which is similar to the study conducted in India [14] and Spain [9]. Likewise, quality of life was significantly associated with economic status and past occupation of the sampled population which is also seen in the study conducted by Aliabadi et al. [20]. Economic status was the most consistent predictor of QOL as shown in the study conducted in Nigeria [24]. Similarly, various other factors seem to contribute to poorer quality of life of elders such as mobility, physical activities, and the presence of chronic illness, which is similar with the findings investigated in the study conducted in Japan [25] and Austria [8]. Likewise, among six facets of QOL, sensory abilities had the highest good scores and social participation had the lowest good scores of quality of life. All the facets except sensory abilities were associated with age group of participants in the present study, and educational status was found to be associated with autonomy, social participation, and intimacy facet of QOL. However, the study shows no association of gender, ethnicity, religion, and past occupation with facets of quality of life.

This study aimed to investigate the association between nutritional status and QOL among the geriatric population of Lahan municipality, and a significant association was found between nutritional status and quality of life with *p* value 0.001 which underline the importance of considering malnutrition when attempting to improve QOL. A similar association was seen in the study conducted in Austria [8]and Iran [9].

## 5. Conclusions

The study showed about one-fifth of the participants had malnutrition. In addition, most of them were at risk of malnutrition which seems to be worsening with the advancing age. The quality of life of the geriatric population was found to be decreasing with their increasing age which explicit those elders are at greater risk to have poor quality of life as their age increases. Nutritional status was significantly associated with overall QOL.

Assessment of geriatric nutritional status helps to detect the malnutrition at an earlier stage, and early corrective interventions can improve their quality of life.

## Figures and Tables

**Table 1:** Distribution of sociodemographic information of participants (*n* = 328).

Characteristics	Frequency (*n*)	Percentage
*Age in years*		
60–67	151	46.0
>67	177	54.0
*Mean* ± *SD* *=* *68.52*±*6.99 years*		

*Sex*		
Male	190	57.9
Female	138	42.1

*Ethnicity*		
Dalit	46	14.1
Disadvantaged Janajatis	43	13.1
Disadvantaged non-Dalit	203	61.9
Religious minorities	28	8.5
Relatively advantaged Janajatis	2	0.6
Upper	6	1.8

*Religion*		
Hindu	298	90.9
Buddhist	3	0.9
Muslims	27	8.2

*Educational status*		
Illiterate	117	35.7
Nonformal	76	23.2
Primary level	71	21.6
Secondary level	59	18.0
Bachelor and above	5	1.5

*Past occupation*		
Agriculture	144	43.9
Business	60	18.3
Job	49	14.9
Wages	29	8.8
Others	46	14.0

**Table 2:** Association between sociodemographic variables and nutritional status.

Variables	Nutritional status			
	Total, *n* (%)	Normal, *n* (%)	At risk/malnourished, *n* (%)	*p* value
*Age*				
60–67	165 (50.3)	104 (63.0)	61 (37.0)	**<0.001**
68 and above	163 (49.7)	9 (5.5)	154 (94.5)

*Gender*				
Male	190 (57.9)	70 (36.8)	120 (63.2)	**0.285**
Female	138 (42.1)	43 (31.2)	95 (68.8)

*Ethnicity*				
Disadvantaged non-Dalit	203 (61.9)	66 (32.5)	137 (67.5)	**0.346**
Others	125 (38.1)	47 (37.6)	78 (62.4)

*Religion*				
Hindu	298 (90.9)	97 (32.6)	201 (67.4)	**0.022**
Others	30 (9.1)	16 (53.3)	14 (46.7)

*Educational status*				
Literate	206 (62.8)	84 (40.8)	122 (59.2)	**0.002**
Illiterate	122 (37.2)	29 (23.8)	93 (76.2)

*Past occupation*				
Paid works	138 (42.1)	57 (41.3)	81 (58.7)	**0.026**
Nonpaid works	190 (57.9)	56 (29.5)	134 (70.5)

^*∗*^ *p* value significant at <0.05.

**Table 3:** Logistic regression on nutritional status with different variables.

Characteristics	Nutritional status	
	Unadjusted	Adjusted
	OR (95% CI)	OR (95% CI)
*Age*		
60–67	Ref.	Ref.
68 and above	**29.17 (13.88–61.31) ** ^*∗∗∗*^	**28.34 (13.35–60.18) ** ^*∗∗∗*^

*Gender*		
Male	Ref.	Ref.
Female	1.29 (0.81–2.05)	1.12 (0.62–2.04)

*Ethnicity*		
Disadvantaged non-Dalit	1.25 (0.78–1.99)	0.88 (0.46–1.68)
Others	Ref.	Ref.

*Religion*		
Hindu	**2.37 (1.11–5.05) ** ^*∗*^	2.26 (0.78–6.56)
Others	Ref.	Ref.

*Educational status*		
Literate	Ref.	Ref.
Illiterate	**2.21 (1.34–3.64) ** ^*∗∗*^	1.83 (0.94–3.57)

*Past occupation*		
Paid works	Ref.	Ref.
Nonpaid works	**1.68 (1.06–2.67) ** ^*∗*^	0.97 (0.51–1.82)

^*∗*^ *p* < 0.05; ^*∗∗*^*p* < 0.01; ^*∗∗∗*^*p* < 0.001; all other statistics are not significant at *p* < 0.05; model adjusted for all covariates.

**Table 4:** Descriptive statistics of transformed score of QOL of geriatric population.

Facets	Total score	Minimum	Maximum	Mean	Standard deviation
Sensory abilities	100	6.25	81.25	49.25	20.28
Autonomy	100	12.50	75.00	37.75	19.06
Past, present, and future abilities	100	6.25	81.25	32.81	20.95
Social participation	100	0.00	75.00	31.40	18.32
Death and dying	100	6.25	75.00	34.79	21.68
Intimacy	100	6.25	81.25	31.00	17.71
Overall QOL	100	6.25	78.12	36.17	19.67

**Table 5:** Quality of life of the participants.

Status	Numbers	Percentage
Good QOL	158	48.2
Poor QOL	170	51.8

**Table 6:** Association between sociodemographic characteristics and domains of quality of life among geriatric population.

Variables	Sensory abilities		Autonomy		Past, present, and future abilities		Social participation		Death and dying		Intimacy		Overall facets	
	*p* value	OR (C.I.)	*p* value	OR (C.I.)	*p* value	OR (C.I.)	p value	OR (C.I.)	*p* value	OR (C.I.)	*p* value	OR (C.I.)	*p* value	OR (C.I.)
Age 0–67/68 and above	0.493	0.85 (0.55–1.329)	**0.001 ** ^*∗*^	0.11 (0.04–0.29)	**0.001 ** ^*∗*^	0.08 (0.03–0.25)	**0.001 ** ^*∗*^	0.06 (0.15–0.28)	**0.001 ** ^*∗*^	0.36 (0.19–0.67)	**0.001 ** ^*∗*^	0.17 (0.06–0.47)	**0.001 ** ^*∗*^	0.07 (0.04–0.12)
Gender Male/ Female	0.098	0.90 (0.43–1.07)	**0.014**	0.40 (0.18–0.84)	0.933	0.97 (0.5–1.88)	0.056	0.43 (0.17–1.04)	0.435	0.80 (0.47–1.38)	0.068	0.59 (0.338–1.04)	0.570	0.88 (0.56–1.36)
Ethnicity Non-Dalit and Janajati/Dalit and Janajati	0.484	0.84 (0.53–1.34)	0.670	1.15 (0.58–2.28)	0.939	1.02 (0.51–2.03)	0.789	1.11 (0.50–2.46)	0.276	1.34 (0.78–2.29)	0.414	0.79 (0.45–1.38)	0.213	1.32 (0.84–2.07)
Religion Hindu/others	0.968	1.01 (0.47–2.16)	0.193	1.87 (0.71–4.91)	0.06	2.36 (0.94–5.92)	0.051	2.20 (0.97–4.98)	0.018	3.13 (1.16–8.47)	0.074	2.06 (0.91–4.65)	0.107	1.86 (0.86–0.4)
Education status Illiterate/ Literate	0.209	1.33 (0.84–2.11)	**0.001 ** ^*∗*^	3.95 (1.61–9.70)	**0.012**	2.71 (1.21–6.09)	**0.001 ** ^*∗*^	3.772 (1.84–7.72)	0.117	1.64 (0.87–3.08)	**0.041 ** ^*∗*^	2.54 (1.01–6.42)	**0.001 ** ^*∗*^	2.39 (1.49–3.84)
Past occupation Nonpaid works/Paid works	0.765	0.935 (0.60–1045)	0.205	1.52 (0.79–2.94)	0.052	1.91 (0.98–3.69)	0.881	1.04 (0.60–1.80)	0.038	1.82 (1.02–3.24)	0.089	1.91 (0.0.89–4.08)	0.104	1.44 (0.92–2.23)

^*∗*^ *p* value significant at <0.05 based on the chi-square test.

**Table 7:** Association between nutritional status and quality of life.

Nutritional status	Quality of life		
	Good QOL, *n* (%)	Poor QOL, *n* (%)	*p* value
Normal nutritional status	103 (91.2)	10 (8.8)	<0.001
At risk of malnutrition	48 (32.0)	102 (68.0)		
Malnourished	7 (10.8)	58 (89.2)		

## Data Availability

Data can be made available upon request.
